# Supplementary material for: Association between oral microbiome and seven types of cancers in East Asian population: a two-sample Mendelian randomization analysis
Source: Front Mol Biosci. 2023 Nov 21;10:1327893. doi: 10.3389/fmolb.2023.1327893 (PMC10702768; doi:10.3389/fmolb.2023.1327893)
Supplement: Supplementary file 1 [file DataSheet1.ZIP › Supplementary Materials/MR plots of saliva/saliva═╝/Pancreatic cancer/pheno.1568_to_pancreatic cancer_forest.pdf]

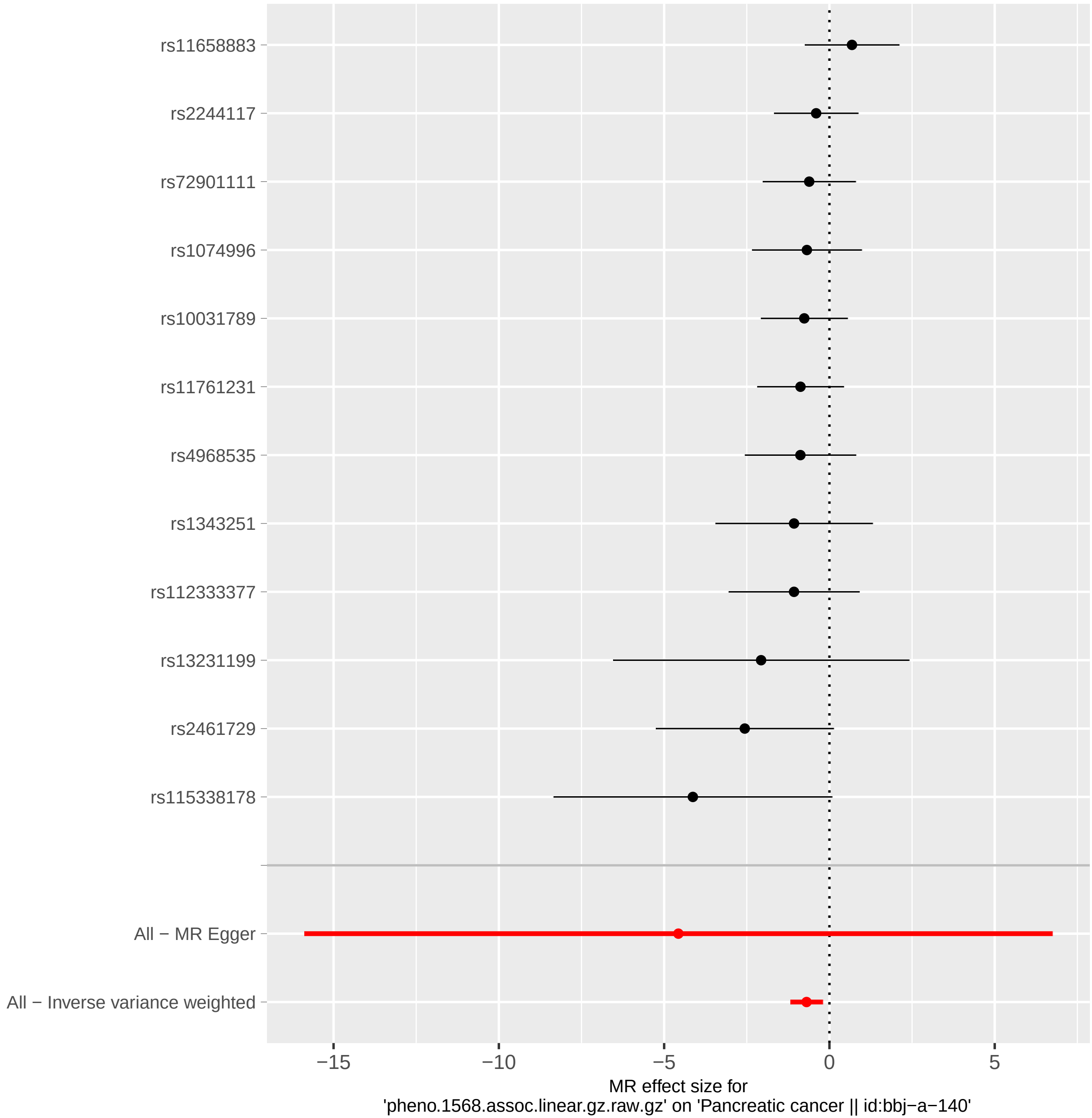
rs11658883
rs2244117
rs72901111
rs1074996
rs10031789
rs11761231
rs4968535
rs1343251
rs112333377
rs13231199
rs2461729
rs115338178
All − MR Egger
All − Inverse variance weighted
−15
−10
−5
0
5
MR effect size for
'pheno.1568.assoc.linear.gz.raw.gz' on 'Pancreatic cancer || id:bbj−a−140'
